# Rheumatoid Arthritis Risk Associated with Periodontitis Exposure: A Nationwide, Population-Based Cohort Study

**DOI:** 10.1371/journal.pone.0139693

**Published:** 2015-10-01

**Authors:** Yin-Yi Chou, Kuo-Lung Lai, Der-Yuan Chen, Ching-Heng Lin, Hsin-Hua Chen

**Affiliations:** 1 Division of Allergy, Immunology and Rheumatology, Department of Internal Medicine, Taichung Veterans General Hospital, Taichung, Taiwan; 2 School of Medicine, National Yang-Ming University, Taipei, Taiwan; 3 School of Medicine, Chung-Shan Medical University, Taichung, Taiwan; 4 Institute of Biomedical Science and Rong Hsing Research Center for Translational Medicine, Chung-Hsing University, Taichung, Taiwan; 5 Department of Medical Education, Taichung Veterans General Hospital, Taichung, Taiwan; 6 Department of Medical Research, Taichung Veterans General Hospital, Taichung, Taiwan; 7 Institute of Public Health and Community Medicine Research Center, National Yang-Ming University, Taipei, Taiwan; 8 Institute of Hospital and Health Care Administration, National Yang-Ming University, Taipei, Taiwan; University of Birmingham, UNITED KINGDOM

## Abstract

**Background:**

The risk of periodontitis (PD) is increased in the patient group of rheumatoid arthritis (RA). RA and PD also shared some pathological mechanism. The aim of this study is to investigate the risk of RA associated with PD exposure.

**Methods and Findings:**

This study identified 3 mutually exclusive cohorts using the 1999–2010 Taiwanese National Health Insurance Research Database (NHIRD) to investigate the association between PD and the risk of incident RA. All patients with PD in 2000 were identified from the database of all enrollees as the PD cohort. From the representative database of 1,000,000 enrollees randomly selected in 2010 (LHID2010), individuals without any periodontal disease (PO) during 1999–2010 were selected as the non-PO cohort. Individuals who were not included in the non-PO cohort and received dental scaling (DS) no more than two times per year during 1999–2010 were selected as the DS cohort from LHID2010. Using cox proportional regression analysis, hazard ratios (HRs) with 95% confidence intervals (Cis) were calculated to quantify the association between PD exposure and RA development. In the three-group comparison using the non-PO cohort as reference, we found that the risk of RA was higher in the PD and DS cohorts (HRs, 1.89 and 1.43; 95% CIs, 1.56–2.29 and 1.09–1.87, respectively). For comparisons between two cohorts, the PD cohort had a higher risk of RA than the non-PO and DS cohorts (HRs, 1.91 and 1.35; 95% CIs, 1.57–2.30 and 1.09–1.67, respectively).

**Conclusion:**

PD was associated with an increased risk of RA development.

## Introduction

Rheumatoid arthritis (RA) is a chronic systemic inflammatory disease with a clinical presentation of polyarthritis that is sometimes accompanied with extra-articular involvement, such as scleritis, vasculitis, and pleuritis. The histological presentation of RA is characterized by chronic synovial inflammation with periarticular osteoporosis and bone erosion. Patients with RA are also associated with a greater risk of cardiovascular disease than patients with diabetes [1]. Although the exact etiology of RA remains unknown, smoking is a well-known risk factor [2]. Many recent studies have focused on the association between periodontitis (PD) and RA development [3–6].

PD is a chronic inflammatory disease triggered by oral bacteria, including *Porphyromonas gingivalis*, *Tannerella forsythia*, and *Treponema denticola*, which are important pathogenic bacteria of PD that can destroy the supporting tissue of teeth, eventually resulting in tooth loss [7]. Moreover, *P*. *gingivalis* is the only microorganism that produces peptidyl-arginine deiminase (PAD) [8]. Some evidence has indicated that many systemic conditions are linked to PD, such as cardiovascular disease, osteoporosis, and type 2 diabetes mellitus (DM) [9].

It is well known that the development of RA is associated with autoimmune responses to citrullinated proteins via the enzymatic activities of PAD [10–12]. PAD expressed by *P*. *gingivalis* (PPAD) is superior to mammalian PAD because of calcium-independent citrullination of C-terminal arginine residues and deaminination of free arginine [8].

A promising hypothesis to interpret the relationship between RA and PD is the autoimmune response to citrullinated proteins produced by PPAD [13]. Previous cross-sectional studies have reported that PD is more prevalent in RA patients [14–18]. Some longitudinal studies have shown that PD exposure was associated with future RA development [19–21]. In vivo studies also revealed a relationship between PD and joint inflammation. Using a collagen-induced arthritis model (CIA), Maresz et al. revealed that *P*. *gingivalis* infection exacerbated CIA and was characterized by greater destruction of bone and cartilage [22]. Furthermore, collagen II has no effect on the development or progression of clinical CIA in animals inoculated with an isogenic PPAD knockout strain. In summary, these observational studies as well as in vivo and in vitro interventional studies indicate that PD and RA may share similar pathological mechanisms, and that the severity of PD may be correlated with the occurrence of and the disease activity of RA.

The Taiwanese National Health Insurance Research Database (NHIRD) is a longitudinal nationwide population-based database that facilitates an epidemiologic study to test the association between PD exposure and RA risk. We have previously completed a case-control study from the NHIRD to show an association between PD exposure and RA development [19]. However, a case-control study design has a lower evidence level than a cohort study design. Here, we conducted a cohort study to estimate the relative risk of incident RA in a nationwide PD cohort compared with the following two cohorts selected from 1 million representative population: individuals without periodontal disease (PO; non-PO cohort) and those who underwent routine dental scaling (DS) without other PD related treatment (DS cohort).

## Methods

### Ethic statement

The study protocol was approved by the Ethics Committee for Clinical Research of Taichung Veterans General Hospital (Taichung, Taiwan). Written informed consent was not obtained because the dataset removed all personal identification information prior to analysis.

### Study design

This study employed a retrospective cohort study design.

### Data source

On March 1, 1995, Taiwan implemented a compulsory National Health Insurance (NHI) Program, which currently covers a population of >99%. The National Health Research Institute manages the NHIRD, which collects data regarding ambulatory care, inpatient services, dental services, traditional Chinese medical services, and drug prescriptions for research purposes. However, demographic information, such as tobacco and alcohol use and examination data, is not included. To improve data accuracy, the Bureau of NHI routinely performs random checks of patient charts [23].

In the present study, the ambulatory, inpatient, and enrollment data of the PD cohort were extracted during 1999–2010 from the NHIRD were used to identify all patients with PD in the year 2000 (fixed cohort). Also, the NHIRD constructed a representative database of 1,000,000 individuals randomly selected from all enrollees who received services in 2010 (LHID2010). The data of the comparison cohorts were extracted from LHID2010. In Taiwan, patients with certain major illnesses, such as cancer and certain autoimmune diseases including RA, are issued a certificate of “catastrophic illness” and are exempt from co-payment. Only those who meet the classification criteria of RA validated by at least two qualified rheumatologists after a thorough review of patients’ medical records, laboratory data, and images are issued a certificate of RA catastrophic illness. The NHIRD includes a catastrophic illness enrollment file for patients with catastrophic illness certificates. In the present study, RA cases were identified from the catastrophic illness enrollment file.

### Definition of PD exposure

The Taiwanese government recommends regular DS one to two times per year to improve dental health. A diagnosis of PD (ICD9-CM Codes 523.3–5) may also be coded by dentists for patients who receive routine dental check-ups and regular DS. Therefore, we defined PD exposure as having at least one ambulatory visit with a diagnosis of PD (ICD9-CM Codes 523.3–5) and concurrently receiving antibiotic therapy or periodontal treatment or DS up to ≥three times in 2000.

### Surrogate measures of PD severity

This study included the number of visits for PD, the cost of PD-related visits, and the receipts for antibiotics or periodontal surgery expenditures in 2000 as surrogate measures of PD severity, assuming that PD severity had a positive correlation with these measures. PD-related cost and number of visits were transformed into bivariates based on the 75^th^ percentile (i.e., 1–92 vs. ≥92 and 1–2 vs. ≥3, respectively). We converted the costs from Taiwan dollars (TWD) to United States dollars (USD) with a conversion rate of 30 TWD to 1 USD. Periodontal surgery comprised gingivectomy, gingivoplasty, periodontal flap surgery, and subgingival curettage.

### Study samples

#### PD cohort from nationwide population

The PD cohort was enrolled from the nationwide Taiwanese population diagnosed with PD in 2000, whereas those diagnosed with RA before that year were excluded.

#### The DS and non-PO cohorts from representative 1 million population

From the LHID2010, after exclusion of individuals born after January 1, 2000 or with a diagnosis of RA before the year 2000, we identified individuals who never had a diagnosis of PO (ICD9-CM Codes 523.x) from 1999 to 2010 as the non-PO cohort, patients who had an ambulatory visit with a diagnosis of PD and received DS concurrently no more than twice per year during 1999–2010 as the DS cohort. Individuals having an ambulatory visit with a diagnosis of PD and who received concurrent periodontal treatment other than DS during 1999–2000 were also excluded from the DS cohort.

### Index date

The index date used in this study was the first day of the observational period. The index date was defined as the first ambulatory visit with a diagnosis of PD (ICD9-CM Codes 523.3–5) in the year 2000 for the PD cohort, while January 1, 2000 was used as the index date for non-PO and DS cohorts.

### Definition of incident RA

RA was defined as at least one ambulatory visit with a diagnosis of RA (ICD9-CM Codes 714.0) followed by the issuing of a catastrophic illness certificate after the index date with no previous history of an ambulatory visit with a diagnosis of RA before the index date.

### Outcome variable

The primary outcome of this study was the time from the index date to the first ambulatory visit with a diagnosis of RA (ICD9-CM Code 714.0) followed by the issuing of a RA catastrophic illness certificate. The censored date was the date of withdrawal from the Taiwanese NHI system for any reason or December 31, 2010, whichever came first.

### Potential confounders

In the present study, age, sex, and DM were considered as potential confounders. Patients who visited an outpatient clinic and were diagnosed with ICD9-CM code 250.x and concurrently received anti-diabetic treatment were defined as having DM.

### Statistical analysis

Analysis of variance was used to compare continuous variables among cohorts, while the chi-square test was used to compare categorical variables. Cox regression analysis was used to calculate the risk of RA development associated with baseline PD status and other potential confounders, as shown by hazard ratios (HRs) with 95% confidence intervals (CIs). The significance of the interaction effects of gender on the association between PD/DM and RA risk was assessed using the Wald test to calculate the probability (*p*) value of the coefficient associated with the product of the indicators of gender and PD/DM. A two-tailed *p* value of <0.05 was considered statistically significant. All statistical analyses were conducted using SPSS version 18.0 for Windows (SPSS, Inc., Chicago, IL, USA).

## Results

A total of 628,628 patients were included in the PD cohort, 168,842 in the non-PO cohort, and 96,542 in the DS cohort. As shown in Table 1, there were significant differences in the mean age and proportion of females among the three cohorts. The DS cohort had the highest proportion of women, while mean age was greatest in the PD cohort. The proportion of patients with a history of DM was higher in the PD cohort than in the DS and non-PD cohorts (3.9% vs. 1.0% and 1.0%, respectively). Among incident RA cases diagnosed with PD in the year 2000 (PD and DS cohorts), the mean time from PD exposure (index date) to RA diagnosis was shorter in the PD cohort than in the DS cohort (4.0 ± 2.8 vs. 4.7 ± 2.9 years). The crude HRs (95% CIs) for RA risk in the PD and DS cohorts were 2.71 (2.24–3.28) and 1.57 (1.20–2.06), respectively, compared with the non-PO cohort.

**Table 1:** Comparison of demographical and clinical data among cohorts.

	PD^a^ (n = 628,628)	DS^a^ (n = 96,542)	Non-PO^a^ (n = 168,842)	*p* value
Female	312,372 (49.7)	51,165 (53.0)	76,358 (45.2)	<0.001
Age*	43.9 ± 17.1	32.1 ± 15.5	27.9 ± 21.3	<0.001
Number of visits for PD^a^ in the year 2000				
1–2	399,154 (63.5)	-	-	
≥ 3	229,474 (36.5)	-	-	
Cost of PD-related visits in the year 2000 (USD^a^)				
1–92	471,152 (74.9)	-	-	
>92	157,476 (25.1)	-	-	
Antibiotic treatment for PD in the year 2000	259,827 (41.3)	-	-	
Periodontal surgery in the year 2000	45,293 (7.2)	-	-	
History of DM^a^	24,354 (3.9)	959 (1.0)	1736 (1.0)	<0.001
RA^a^	1,110 (0.2)	96 (0.1)	117 (0.1)	<0.001
Person-years	4,737,514	725,823	1,407,955	
Incidence of RA^a^ per 10^5^ person-years	23.4	13.2	8.3	

Results are presented as number (%), unless otherwise indicated.

*Presented as mean ± SD.

^a^Abbreviations: DM, diabetes mellitus; DS, dental scaling; PD, periodontitis; PO, periodontal disease; RA, rheumatoid arthritis; USD, United States Dollar.

**Fig 1:**
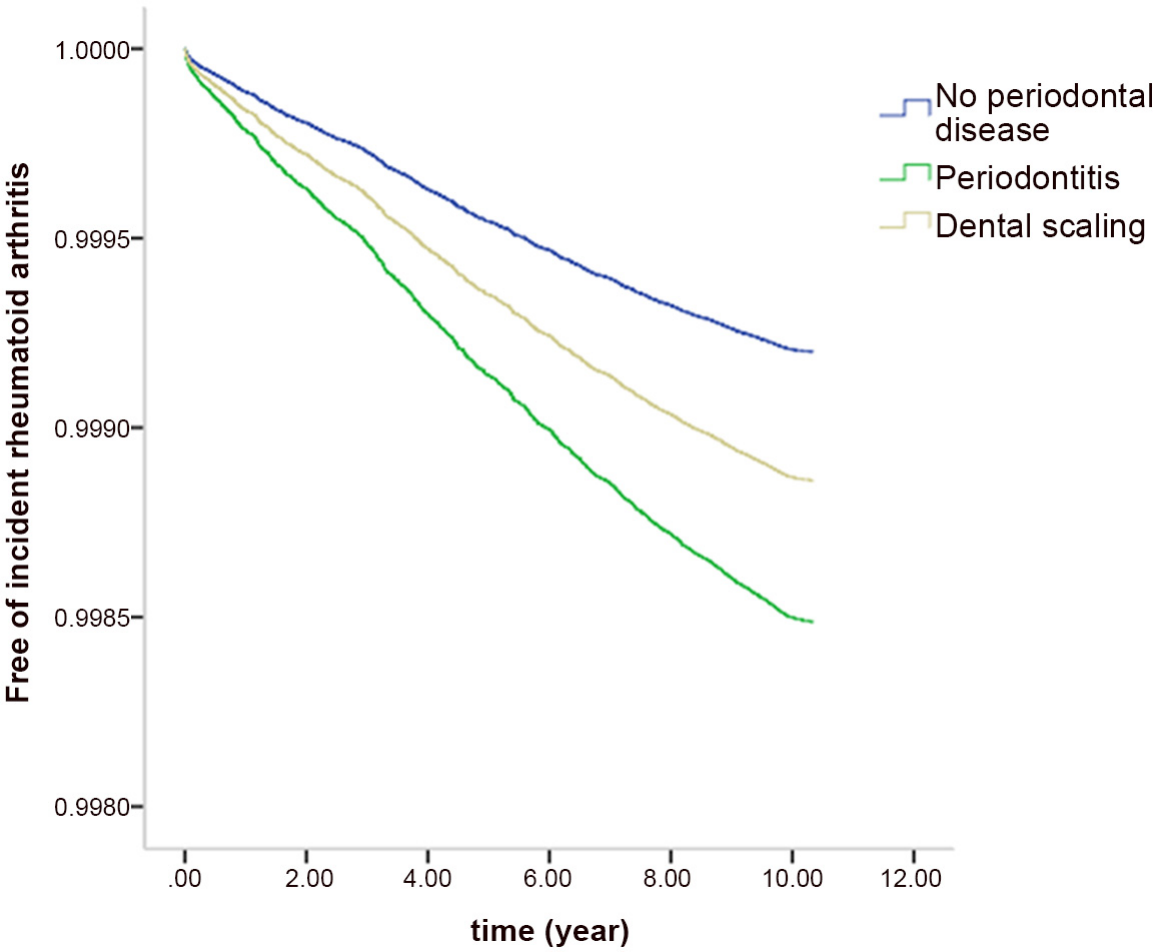
Free of incident rheumatoid arthritis rates among individuals without periodontal diseases, patients with periodontitis, and patients receiving dental scaling.

As shown in Table 2, after adjusting for age, sex, and DM history, the risk of RA development was higher in PD and DS cohorts (HR, 1.89 and 1.43; 95% CI, 1.56–2.29 and 1.09–1.87, respectively) than in the non-PO cohort. Using the DS cohort as the reference cohort, the adjusted HRs (95% CIs) of RA risk for the PD and non-PO cohorts were 1.33 (1.08–1.64) and 0.70 (0.54–0.92), respectively. There were no significant differences in the associations between periodontal status and RA risk between men and women, although women had a higher risk of RA development (HR, 3.62; 95% CI, 3.19–4.11) than men. A history of treated DM was associated with a lower risk of RA development (HR, 0.65; 95% CI, 0.49–0.88), and this protective effect seemed stronger in men (HR, 0.55; 95% CI, 0.28–1.07). However, the effect of sex on the association between DM and RA risk was not statistically significant (interaction, *p* = 0.552). Fig 1 illustrates the rates of incident RA among the three cohorts. A survival curve for incident RA of the three cohorts is shown in Fig 1.

**Table 2:** Adjusted HRs with 95% CIs for the association between variables and RA development.

Variable	Female	Male	Total
Periodontal status^#^			
Non-PO^a^	1.00 (reference)	1.00 (reference)	1.00 (reference)
DS^a^	1.47 (1.08–2.00)	1.29 (0.72–2.32)	1.43 (1.09–1.87)
PD^a^	1.96 (1.57–2.45)	1.69 (1.15–2.48)	1.89 (1.56–2.29)
Female	-	-	3.62 (3.19–4.11)
Age	1.04 (1.03–1.04)	1.04 (1.03–1.04)	1.03 (1.03–1.03)
DM* ^a^	0.68 (0.49–0.95)	0.55 (0.28–1.07)	0.65 (0.49–0.88)

Adjusted variables include sex, age, and history of DM requiring medication.

^#^Interaction effect of gender on the association between periodontal status and RA risk; *p* = 0.618.

*Interaction effect of gender on the association between diabetes mellitus and RA risk; *p* = 0.552.

^a^Abbreviations: DM, diabetes mellitus; PD, periodontitis; DS, dental scaling.

As shown in Table 3, the PD cohort had a higher risk of RA development regardless of using the non-PO or DS cohorts for comparison.

**Table 3:** Adjusted HRs with 95% CIs for RA risk in patients with PD compared with various cohorts.

Comparison cohort	HR^a^ (95% CI)	*p* value
Non-PO^a^	1.90 (1.57–2.30)	<0.001
DS^a^	1.35 (1.09–1.67)	0.005

Adjusted variables included sex, age, and a history of DM requiring medication.

^a^Abbreviations: DS, dental scaling; PO, periodontal disease; HR, hazards ratio.

As shown in Table 4, the risk of incident RA was higher in patients with higher number of PD-related visits, those with a higher cost of PD-related visits, and those who received periodontal surgery than those without periodontal disease.

**Table 4:** Adjusted HRs with 95% CIs for the association between history of PD and RA development involving individuals without periodontal disease for comparison.

Variable	Female	Male	Total
Number of visits for PD^a^ in the year 2000			
No periodontal disease	1.00 (reference)	1.00 (reference)	1.00 (reference)
1–2	1.93 (1.54–2.43)	1.54 (1.03–2.30)	1.83 (1.50–2.23)
≥3	2.02 (1.59–2.57)	1.99 (1.32–2.99)	2.02 (1.64–2.48)
Cost of PD-related visits in the year 2000 (USD^a^)			
No periodontal disease	1.00 (reference)	1.00 (reference)	1.00 (reference)
<92	1.94 (1.55–2.42)	1.63 (1.10–2.41)	1.86 (1.53–2.26)
≥92	2.06 (1.60–2.64)	1.96 (1.28–3.02)	2.03 (1.64–2.52)
Antibiotic treatment for PD in the year 2000			
No periodontal disease	1.00 (reference)	1.00 (reference)	1.00 (reference)
No	1.98 (1.58–2.49)	1.77 (1.19–2.64)	1.93 (1.03–1.04)
Yes	1.94 (1.53–2.46)	1.64 (1.09–2.47)	1.86 (1.52–2.29)
Periodontal surgery in the year 2000			
No periodontal disease	1.00 (reference)	1.00 (reference)	1.00 (reference)
No	1.91 (1.53–2.39)	1.74 (1.18–2.56)	1.87 (1.54–2.27)
Yes	2.59 (1.91–3.49)	1.43 (0.78–2.59)	2.28 (1.74–2.97)

Adjusted variables included sex, age, and history of DM requiring medication.

^a^Abbreviations: DM, diabetes mellitus; PD, periodontitis

## Discussion

This is the first nationwide population-based cohort study to assess the association between PD exposure and RA risk using the NHIRD. The main finding of our study demonstrated a higher risk of RA in patients with PD exposure than those who never received a diagnosis of PD, and those who received DS. A dose–response relationship between PD and RA risk was also observed when PD-related utilizations were used as the proxy measures of PD severity. Consistent with the findings of two recent case-control studies [19, 21] and one population-based diabetic cohort study [20], the results of this cohort study suggested that PD exposure is associated with a greater risk of RA development. In our previous case-controlled study, the risk of RA associated with PD exposure (odds ratio, 1.16; 95% CI, 1.12–1.20) was lower than that estimated in this study. Possible explanations of this inconsistency include differences in study designs and definitions of patients without periodontitis. In our previous case-controlled study, we may have misclassified those who received DS as patients without PD exposure, and thereby underestimated the association between PD and RA risk. We hypothesized that some of the individuals who received DS up to one to two times per year with concurrent PD coding may have had mild PD, rather than visiting dentists for routine dental examinations. In this study, the findings that the risk of RA in patients who received DS was higher than that in patients without PO but lower than that in PD patients support our hypothesis. In contrast to the findings of our study, in the results of another large longitudinal study using a cohort of American females no significant association between PD exposure and RA risk was found [24]. However, the use of periodontal surgery and/or tooth loss to define PD exposure may lead to the misclassification of individuals with mild PD as non-PD individuals; therefore, the risk of RA associated with PD may be underestimated. Consistent with our previous case-control study [19], the findings of the present cohort study also demonstrated that patients with a history of DM were at a lower risk of RA development.

The strength of this study was the use of a nationwide database composed of Taiwanese men and women, which may have avoided selection bias; thus, it is applicable to the general population in Taiwan. The cohort study design per se also confers a higher level of evidence to suggest a causal relationship rather than the case-controlled design, as in our previously study [19].

There were some limitations to this study. First, there was no data regarding smoking status, which is an important confounding factor. However, these results demonstrated a significant association between PD and RA in non-smoking individuals [21]. In addition, if the association between PD and RA development was driven by smoking, this association should be stronger in the population with a higher prevalence of tobacco use. Given the marked higher prevalence of tobacco use in men than in women (65.7% vs. 5.4%) based on our previous survey [25], the association between PD and RA was less likely to be driven solely by the unmeasured smoking status because this study showed a higher magnitude of this association in women than in men. Second, the accuracy of PD diagnoses based on administrative data was a concern. However, such non-differential misclassification bias related to PD exposure is always biased toward the null. In addition, the accuracy of RA diagnoses was not a concern because the issuing of a catastrophic illness certificate was made only after the validation of RA diagnosis by two or more rheumatologists via the comprehensive review of clinical data. Third, potential detection bias can occur if the PD and/or DS cohort had more medical visits than the non-PO cohort. This type of a detection bias may lead to an earlier detection of RA than that for the non-PO cohort, resulting in an overestimation of RA risk in the PD or DS cohort [26]. On the contrary, an underestimation of RA risk in the PD or DS cohort may occur if some patients with PD were misclassified as non-PD because they sought medical treatment that was not covered by NHI (for instance, use of over-the-counter drugs). Finally, laboratory and clinical information about PD severity and the association between PD and anti-cyclic citrullinated peptide antibody levels was not collected in the database.

## Conclusion

The findings of this nationwide, population-based cohort study revealed a significant association between PD exposure and RA risk. However, further prospective, population-based cohort studies are needed to confirm this association and to identify other related genetic and environmental risk factors for RA development.

## Supporting Information

S1 Dataset Dataset of the PD, DS and non-PO cohorts. (RAR)
